# The Synergistic Effects of Orthokeratology and Atropine in Slowing the Progression of Myopia

**DOI:** 10.3390/jcm7090259

**Published:** 2018-09-07

**Authors:** Lei Wan, Chang-Ching Wei, Chih Sheng Chen, Ching-Yao Chang, Chao-Jen Lin, Jamie Jiin-Yi Chen, Peng-Tai Tien, Hui-Ju Lin

**Affiliations:** 1 School of Chinese Medicine, China Medical University, Taichung 404, Taiwan; 2 Department of Biotechnology, Asia University, Taichung 413, Taiwan; cychang@asia.edu.tw; 3 Department of Obstetrics and Gynecology, China Medical University Hospital, Taichung 404, Taiwan; 4 Research Center for Chinese Medicine & Acupuncture, China Medical University, Taichung 404, Taiwan; 5 Children’s Hospital, China Medical University Hospital, Taichung 404, Taiwan; weilonger@gmail.com; 6 College of Medicine, China Medical University, Taichung 404, Taiwan; u702054@hotmail.com; 7 Division of Chinese Medicine, Asia University Hospital, Taichung 413, Taiwan; pluto915@mail2000.com.tw; 8 Department of Pediatrics, Changhua Christian Children’s Hospital, Changhua 500, Taiwan; 124140@cch.org.tw; 9 School of Medicine, Chung Shan Medical University, Taichung 402, Taiwan; 10 Department of Ophthalmology, China Medical University Hospital, Taichung 404, Taiwan; jane.editing@gmail.com; 11 Graduate Institute of Clinical Medical Science, China Medical University, Taichung 404, Taiwan

**Keywords:** myopia, orthokeratology, atropine

## Abstract

Atropine and orthokeratology (OK) are both effective in slowing the progression of myopia. In the current study, we studied the combined effects of atropine and OK lenses on slowing the progression of myopia. This retrospective study included 84 patients who wore OK lenses and received atropine treatment (OA) and 95 patients who wore OK lenses alone (OK) for 2 years. We stratified patients into low (<6 D, LM) and high (≥6 D, HM) myopia groups, as well as two different atropine concentrations (0.125% and 0.025%). Significantly better LM control was observed in OA1 patients, compared with OK1 patients. Axial length was significantly shorter in the OA1 group (24.67 ± 1.53 mm) than in the OK1 group (24.9 ± 1.98 mm) (*p* = 0.042); similarly, it was shorter in the OA2 group (24.73 ± 1.53 mm) than in the OK2 group (25.01 ± 1.26 mm) (*p* = 0.031). For the HM patients, OA3 patients compared with OK3 patients, axial length was significantly shorter in the OA3 group (25.78 ± 1.46 mm) than in the OK3 group (25.93 ± 1.94 mm) (*p* = 0.021); similarly, it was shorter in the OA4 patients (25.86 ± 1.21 mm) than in the OK4 patients (26.05 ± 1.57 mm) (*p* = 0.011). Combined treatment with atropine and OK lenses would be a choice of treatment to control the development of myopia.

## 1. Introduction

Myopia is one of the major global causes of visual impairment and has an extensive impact on public health care systems and economies worldwide [1]. Approximately 153 million individuals over the age of 5 years exhibit some distance visual impairment; 8 million of these individuals are effectively blind because of uncorrected refractive errors [2]. In Taiwan, the prevalence of myopia in 6-year-old children is 9.4% and reaches > 75% in 15-year-old adolescents. This prevalence increases to 80–90% in 18-year-olds; 10–20% of individuals in this age group are highly myopic [1]. High myopia is a major cause of blindness because of its association with retinal detachment [3], macular choroidal degeneration [4,5], premature cataracts [5,6], and glaucoma [7]. In patients with myopia ≥6 D, the annual incidence of retinal detachment is estimated at 3.2%. High myopia also increases the risk of macular choroidal neovascularization, which is up to 9-fold higher in patients with myopia ≥6 D [1,8].

Atropine is a non-selective mAChR antagonist. Animal studies of myopia have shown that atropine effectively prevents the axial elongation that contributes to myopia [9,10]. Atropine has been shown to inhibit myopia progression in the tree shew [11], monkey, chick, and mouse. In contrast to the eyes of mammals, chick (avian) eyes consist of striated intraocular muscle; therefore, atropine cannot exert mydriatic or cycloplegic effects in chick eyes, which indicates a nonaccommodative mechanism for atropine in slowing myopia progression [11,12,13,14,15,16,17]. Moreover, human clinical trials have shown the effectiveness of daily atropine administration in reducing myopia progression [9,18]. However, it has been reported that there are rebound effects after discontinuation of atropine, where eyes treated with atropine exhibited higher myopia progression rates, compared with eyes treated with placebo. Nevertheless, after atropine treatment, myopia progression is significantly lower in the atropine-treated group [19,20,21,22,23,24,25].

Orthokeratology (OK) lenses comprise another successful treatment approach to controlling myopia progression. OK lenses are custom-designed rigid contact lenses that reshape the cornea to reduce refractive error. Wearing OK lenses overnight reduces the need for patients to wear contact lenses or spectacles in the daytime [26,27,28,29,30]. However, OK lenses can cause higher-order aberrations and lowered contrast sensitivity [31,32]. Regardless of the side effects elicited by OK lenses, the use of OK lenses for control of myopia is increasing.

In our previous report, we compared the effectiveness of OK lenses and atropine in controlling the refractive error of myopia. We found slightly better myopia control using overnight OK lenses, compared with 0.125% atropine [33]. In the present study, we aimed to determine whether the effects of atropine and OK lenses can combine to slow the progression of myopia compare to OK lenses alone.

## 2. Patients and Methods

### 2.1. Patients

**Figure 1:**
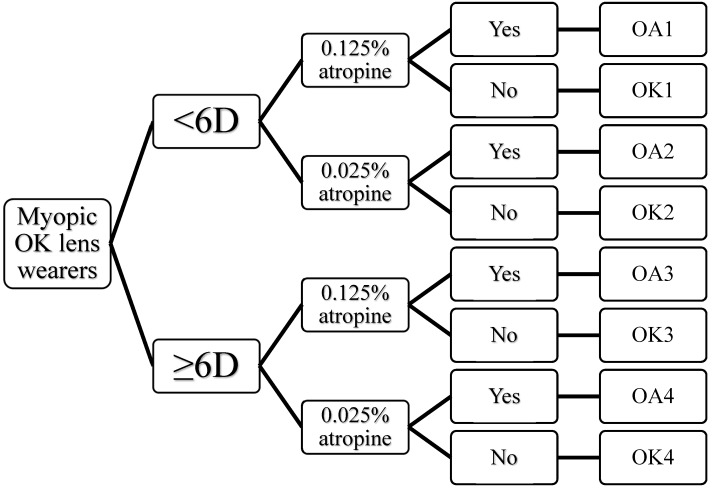
Treatments and groupings of subjects (OK—orthokeratology; OA—orthokeratology + atropine).

This retrospective cohort study was undertaken for a total of 2 years. Patients were treated with OK lenses and treated with/without atropine (Figure 1). Patients with regular examinations, who had complete clinical data during the study period (2 years, from 2014 to 2016) were included in this study. Myopia patients treated with 0.125% atropine were collected from July 2014 to August 2014 and those treated with 0.025% atropine were collected from November 2014 to December 2014. It is known that the myopia progression is slower in summer than in winter [34,35,36], therefore, we enrolled different groups of patients to evaluate the efficacy of OA and OK. All patients had a visual acuity with near and distance correction of 0.01 logMAR (20/20) or better, as determined by the Landolt CETDRS Distance Chart. Best-corrected visual acuity (BCVA) measurements were performed between 2 and 4 o’clock in the afternoon. Patients with retinopathy, prematurity, neonatal problems, history of genetic disease, connective tissue diseases (e.g., Strickler or Marfan syndromes), organic eye diseases, or intraocular surgery were excluded. Patients with differences of refractive error > 2 D between their eyes were also excluded. Inclusion criteria were patients, ages 7–17 years old, with distance corrected acuity of 0.01 logMAR (20/20) or better. Myopic spherical equivalences of 1.5–5.75 D were grouped as myopia <6 D, spherical equivalent of 6–7.5 D were grouped into myopia ≥6 D. Comprehensive ophthalmologic examinations, including visual acuity, refraction error, slit lamp examination, ocular movements, intraocular pressure, and fundoscopy were performed before treatment (baseline) and at every three months. All patients were followed for 24 months.

### 2.2. Treatments

Two concentrations of atropine 0.125% and 0.025% concentrations of atropine (Wu-Fu Pharmaceutical CC., Inc., YiLan, Taiwan) were used. Since there is no commercially available formulation of low concentration atropine (0.01%) in Taiwan, the preparation of 0.025% atropine was performed in a laminar flow cabinet. Patients self-selected treatment with OK lens alone or a combined treatment of OK lens and atropine. The advantages and disadvantages of the two different treatments were explained to the patients and their guardians. Patients treated with atropine received one drop of atropine every night, 30 min before sleep, and were included in this study only if they did not discontinue the atropine for >10 days. OK lenses were inserted 1 h after the administration of atropine. We used four-zone, reverse-geometry lenses (Emerald Lenses; Euclid systems Corp., Herndon, VA, USA, manufactured from Boston XO material; Polymer Technology Corp., Wilmington, MA, USA), which has a nominal Dk of 100 × 10^11^ cm^2^/s (mL O_2_/mL mmHg). The lenses have a nominal central thickness of 0.22 mm, with a diameter of 10.4–11 mm. For patients with refractive error greater than 5.75 D, OK lenses with double reverse curves and a dual geometric design were used. OK lenses were changed when the uncorrected visual acuity reached <0.3 logMAR. Patients were required to wear the OK lenses for at least 6–8 h. Ophthalmologic examinations were performed every 3 months, including slit lamp examinations to evaluate the potential side effects.

### 2.3. Ophthalmologic Examinations

Non-cycloplegic and cycloplegic refraction, as well as axial length, were determined before the initiation of atropine or OK lens treatments (baseline); the differences of refraction and axial length after 2 years of treatment were compared. Refractive data were determined by autorefractor/auto-keratometer (ARK 700A; Nikon, Tokyo, Japan) by trained optometrists in accordance with the study protocol. The average spherical equivalent between the left and right eyes for each patient were used for the analysis. Axial lengths were evaluated by a noncontact optical biometric device by using the average of five successive measurements (IOL Master; Carl Zeiss Meditec AG, Jena, Germany). The mean axial length of left and right eyes of each patient was used in the analysis. The Mann-Whitney U test was used to compare differences in refractive error and axial length. A *p*-value < 0.05 was regarded as statistically significant.

## 3. Results

### 3.1. Patients with Myopia <6 D

Twenty patients who were treated with OK lens + 0.125% atropine (OA1 group) and 26 patients treated with OK lens only (OK1 group) were enrolled. The average age of the patients was 10.6 ± 1.2 years for the OA1 group and 10.2 ± 1.7 years for OK1 group. The female/male ratio in both groups is equal to 1. The average axial length at baseline for the OA1 group was 24.12 ± 1.28 mm and for the OK1 group was 24.32 ± 1.53 mm. The average spherical equivalent at baseline for the OA1 group was 4.28 ± 1.75 D and for the OK1 group is 4.25 ± 1.25 D. After 2 years, axial length was significantly shorter in the OA1 group (24.67 ± 1.53 mm) than in the OK1 group (24.9 ± 1.98 mm) (*p* = 0.042). We found a significant better myopia control in OA1 than OK1 (*p* = 0.022). Moreover, average spherical equivalent was also significantly lower in the OA1 group (4.75 ± 0.75 D) than in the OK1 group (4.8 ± 0.5 D) (*p* = 0.041). We found no differences between groups in accommodation, photopic pupil diameter, or mesopic pupil diameter at baseline; all were significantly different (all *p* < 0.001) after 2 years, due to the mydriasis and cycloplegia side effects of atropine. There was no difference in BCVA (Table 1).

The combined effects of low concentration atropine (0.025%) and OK lens were also evaluated. Twenty patients who were treated with OK lens + 0.025% atropine (OA2 group) and 20 patients who were treated with OK lens only (OK2 group). The average age of the patients was 10.4 ± 1.3 years for the OA2 group and 10.3 ± 1.4 years for the OK2 group. The female/male ratio in both groups is equal to 1. The average axial length at baseline for the OA2 group was 24.08 ± 1.31 mm and for the OK2 group was 24.19 ± 1.24 mm. The average spherical equivalent at baseline for the OA2 group was 4.53 ± 1.23 D and for the OK2 group was 4.63 ± 1.35 D. After 2 years, axial length was significantly shorter in the OA2 group (24.73 ± 1.53 mm) than in the OK2 group (25.01 ± 1.26 mm) (*p* = 0.031). We found a significant better myopia control in OA2 than OK2 (*p* = 0.029). The average spherical equivalent was also significantly lower in the OA2 group (4.83 ± 1.12 D) than in the OK2 group (5.13 ± 1.56 D) (*p* = 0.039). We found no differences in accommodation, photopic pupil diameter, or mesopic pupil diameter at baseline; all were significantly different (all *p* < 0.001) after 2 years, due to the mydriasis and cycloplegia side effects of atropine. There was no difference in BCVA (Table 1).

### 3.2. Patients with Myopia ≥6 D

We enrolled 24 patients who were treated with OK lens + 0.125% atropine in the OA3 group and 29 patients in the OK3 group. The average age of the patients was 11.0 ± 1.8 years for the OA3 group and 10.8 ± 1.8 years for the OK3 group. The average axial length at baseline for the OA3 group was 25.21 ± 1.35 mm and for the OK3 group was 25.29 ± 1.78 mm. The female/male ratio in OK3 is 1.07 while others are equal to 1. The average spherical equivalent at baseline for the OA3 group was 6.75 ± 1.5 D and for the OK3 group was 6.75 ± 1.5 D. After 2 years, axial length was significantly shorter in the OA3 group (25.78 ± 1.46 mm) than in the OK3 group (25.93 ± 1.94 mm) (*p* = 0.021). We found a significant better myopia control in OA3 than OK3 (*p* = 0.015). The average spherical equivalent was also significantly lower in the OA3 group (7.0 ± 0.5 D) than in the OK3 group (7.2 ± 0.75 D) (*p* = 0.028). We found no differences in accommodation, photopic pupil diameter, or mesopic pupil diameter at baseline; all were significantly different (all *p* < 0.001) after 2 years, due to the mydriasis and cycloplegia side effects of atropine. There was no difference in BCVA (Table 2).

For patients with spherical equivalent ≥6 D, we enrolled 20 patients in the OA4 group and 20 patients in the OK4 group. The average age of the patients was 12.21 ± 1.63 years for the OA4 group and 12.78 ± 2.32 years for the OK4 group. The female/male ratio in both groups is equal to 1. The average spherical equivalent at baseline for the OA4 group was 6.63 ± 1.56 D and for the OK4 group was 6.67 ± 1.73 D. The average axial length at baseline for the OA4 group was 25.28 ± 1.53 mm and for the OK4 group was 25.65 ± 1.67 mm. After 2 years, axial length was significantly shorter in the OA4 group (25.86 ± 1.21 mm) than in the OK4 group (26.05 ± 1.57 mm) (*p* < 0.011). The average spherical equivalent was also significant lower in the OA4 group (7.12 ± 1.83 D) than in the OK4 group (7.32 ± 1.87 D) (*p* < 0.027). We found no differences in accommodation, photopic pupil diameter, or mesopic pupil diameter at baseline; all were significantly different (all *p* < 0.001) after 2 years, due to the mydriasis and cycloplegia side effects of atropine. There was no difference in BCVA (Table 2).

High Order Aberration was also checked in every examination. Coma ($Z_{3}^{1}$, $Z_{3}^{-1}$) and spherical aberration ($Z_{4}^{0}$) was compared between the groups. There is only a significant difference noted in spherical aberration (myopia ≥6 D) in patients with and without atropine (*p* = 0.038) (Table 2). There are no other significant differences noted in other comparisons.

## 4. Discussion

Several optical and pharmacological treatments to prevent myopia have shown promising effects. To improve the efficacy of therapy against myopia, the combined effects of the two most effective treatments, atropine, and OK lenses, were evaluated. We found improving myopia control by combining OK lenses with either 0.125% or 0.025% atropine, compared with OK lenses alone.

The specific mechanism of how OK lenses reduce myopia progression remains unknown. Two theories have been proposed: (1) the peripheral myopic defocus theory, in which OK lenses cause peripheral myopic defocus at the horizontal and vertical meridians, thus controlling myopia progression; (2) Increasing accommodation improves binocular vision, in which OK lenses improve the accommodative function (i.e., reduce accommodative lag) of myopic eyes thus controlling myopia progression.

The potential mechanism on the combined effect of atropine treatment and OK lenses is that large pupil diameter increased retinal illumination which would lower the myopic shift in the peripheral retina and enhance the effect of OK lens to slow axial growth in myopia. Light cycle is closely related to eye growth and maturation [37]. However, large pupil in atropine group may be not the only solution, since the pupils of high myopic patients are slightly small then low myopia patients in our study. Another possible explanation is that increasing positive high order aberration (HOA) in the OA groups. Previous study revealed that longer axial length having fewer positive values of fourth order and root mean square spherical aberration [38,39]. The increase of myopia decreases spherical aberration. The third reason is that the change of accommodation. Previous study had proved that myopic control would be more beneficial to lower amplitude of accommodation children than that to higher amplitude of accommodation children in OK lens users [40]. OK lens enhance accommodation and provides some basis for slowing myopia progression. In our study, OA patients were all with decreased accommodation. These conditions are more dominant in high myopia group ≥6 D. These support the theory of the interaction of atropine, accommodation, and OK lenses.

A limitation of this study was that we only checked patients’ refractive error (after discontinuation of OK lenses for 10 days) other than axial length every 6 months. Patients must endure the inconvenience of discontinuation of the OK lenses. Moreover, most patients who used OK lenses exhibited overall good vision; thus, patients and families might forget the important of using atropine, making it highly necessary to promote the importance of using atropine in addition to the OK lenses in the combined OK lens and atropine treatment group. The patients or their guardians chose to use atropine only or to accept the combinational therapy, which would potentially cause difference in the effectiveness of myopia control since the expenses of purchasing OK lenses are not covered by the national health insurance in Taiwan. We did not have their occupation, education or family income statuses of their parents which may potentially cause bias in selecting the treatments. Readers should take this issue into consideration when they read this paper.

## 5. Conclusions

Atropine and OK lenses involve completely different mechanisms to slow myopia progression; hence, together they provide improving control of myopia progression than either atropine or OK lenses alone. In the present study, we provide the clinical evidence that combined treatment with atropine and OK lenses achieves a slightly better control of myopia progression.

## Figures and Tables

**Table 1:** The effect of 0.125% and 0.025% atropine on orthokeratology (OK)-treated patients with spherical equivalent <6 D.

	Atropine (0.125%)		*p*-Value	Atropine (0.025%)		*p*-Value
	Yes (OA1) (*N* = 20)	No (OK1) (*N* = 26)		Yes (OA2) (*N* = 20)	No (OK2) (*N* = 20)	
**Age**	10.6 ± 1.2	10.2 ± 1.7	>0.05	10.4 ± 1.3	10.3 ± 1.4	>0.05
**Female: male ^#^**	1:1	1:1		1:1	1:1	
**Axial length (mm)**						
Baseline	24.12 ± 1.28	24.32 ± 1.53	>0.05	24.08 ± 1.31	24.19 ± 1.24	>0.05
2 years	24.67 ± 1.53	24.9 ± 1.98	0.042	24.73 ± 1.53	25.01 ± 1.26	0.031
Difference in axial length	0.55 ± 0.12	0.58 ± 0.09	0.022	0.65 ± 0.18	0.83 ± 0.16	0.029
**Spherical equivalent (D)**						
Baseline	4.25 ± 1.75	4.25 ± 1.25	>0.05	4.53 ± 1.23	4.63 ± 1.35	>0.05
2 years	4.75 ± 0.75	4.8 ± 0.5	0.041	4.83 ± 1.12	5.13 ± 1.56	0.039
**Accommodation**						
Baseline	16.2 ± 3.1	16.7 ± 3.4	>0.05	16.3 ± 3.2	16.5 ± 3.4	>0.05
2 years	4.2 ± 2.7	16.3 ± 3.2	<0.001	4.6 ± 1.56	16.4 ± 3.2	<0.001
**Photopic pupil diameter**						
Baseline	3.8 ± 0.4	3.7 ± 0. 5	>0.05	3.9 ± 0.6	3.7 ± 0.45	>0.05
2 years	6.8 ± 0.5	3.6 ± 0.4	<0.001	6.2 ± 0.6	3.6 ± 0.5	<0.001
**Mesopic pupil diameter**						
Baseline	4.7 ± 0.5	4.3 ± 0.5	>0.05	4.7 ± 0.4	4.6 ± 0.6	>0.05
2 years	7.2 ± 0.4	4.6 ± 0.6	<0.001	6.6 ± 0.7	4.7 ± 0.5	<0.001
**Distance BCVA (log MAR)**						
Baseline	0.01 ± 0.01	0.01 ± 0.01	>0.05	0.01 ± 0.00	0.01 ± 0.01	>0.05
2 years	0.01 ± 0.01	0.01 ± 0.01	>0.05	0.01 ± 0.01	0.01 ± 0.01	>0.05
**Near BCVA (log MAR)**						
Baseline	0.01 ± 0.00	0.01 ± 0.01	>0.05	0.01 ± 0.00	0.01 ± 0.01	>0.05
2 years	0.12 ± 0.02	0.01 ± 0.01	>0.05	0.02 ± 0.02	0.01 ± 0.01	>0.05

^#^ Patients were treated with orthokeratology lenses, with or without atropine treatment for 2 years.

**Table 2:** The effect of 0.125% and 0.025% atropine on orthokeratology (OK)-treated patients with spherical equivalent ≥6 D.

	Atropine (0.125%)		*p*-Value	Atropine (0.025%)		*p*-Value
	Yes (OA3) (*N* = 24)	No (OK3) (*N* = 29)		Yes (OA4) (*N* = 20)	No (OK4) (*N* = 20)	
**Age**	11.0 ± 1.8	10.8 ± 1.8	>0.05	10.8 ± 1.2	10.9 ± 1.3	>0.05
**Female: male ^#^**	1:1	1.07:1		1:1	1:1	
**Axial length (mm)**						
Baseline	25.21 ± 1.35	25.29 ± 1.78	>0.05	25.28 ± 1.53	25.65 ± 1.67	>0.05
2 years	25.78 ± 1.46	25.93 ± 1.94	0.021	25.86 ± 1.21	26.05 ± 1.57	0.011
Difference in axial length	0.57 ± 0.17	0.64 ± 0.14	0.015	0.58 ± 0.08	0.4 ± 0.15	0.023
**Spherical equivalent (D)**						
Baseline	6.75 ± 1.5	6.75 ± 1.5	>0.05	6.63 ± 1.56	6.67 ± 1.73	>0.05
2 years	7.0 ± 0.5	7.2 ± 0.75	0.028	7.12 ± 1.83	7.32 ± 1.87	0.027
**Accommodation**						
Baseline	16.6 ± 2.9	16.8 ± 3.2	>0.05	16.6 ± 2.8	16.8 ± 3.1	>0.05
2 years	3.8 ± 2.9	15.9 ± 3.8	<0.001	3.9 ± 2.01	16.6 ± 2.9	<0.001
**Photopic pupil diameter**						
Baseline	3.9 ± 0.5	3.8 ± 0.7	>0.05	3.8 ± 0.57	3.6 ± 0.63	>0.05
2 years	6.6 ± 0.4	3.5 ± 0.6	<0.001	6.0 ± 0.7	3.7 ± 0.5	<0.001
**Mesopic pupil diameter**						
Baseline	4.8 ± 0.6	4.5 ± 0.7	>0.05	4.8 ± 0.5	4.7 ± 0.6	>0.05
2 years	6.9 ± 0.6	4.5 ± 0.8	<0.001	6.8 ± 0.6	4.8 ± 0.5	<0.001
**Distance BCVA (log MAR)**						
Baseline	0.01 ± 0.01	0.01 ± 0.01	>0.05	0.01 ± 0.01	0.01 ± 0.00	>0.05
2 years	0.01 ± 0.01	0.01 ± 0.01	>0.05	0.01 ± 0.00	0.01 ± 0.00	>0.05
**Near BCVA (log MAR)**						
Baseline	0.00 ± 0.01	0.01 ± 0.01	>0.05	0.01 ± 0.01	0.01 ± 0.00	>0.05
2 years	0.02 ± 0.01	0.01 ± 0.01	>0.05	0.012 ± 0.03	0.01 ± 0.00	>0.05

^#^ Patients were treated with orthokeratology lenses, with or without atropine treatment for 2 years.
